# Clinical Lectures on Diseases of the Eye

**Published:** 1864-09

**Authors:** E. L. Holmes

**Affiliations:** Chicago, Lecturer on Diseases of the Eye and Ear in Rush Medical College, and Surgeon of the Chicago Charitable Eye and Ear Infirmary


### CLINICAL LECTURES ON DISEASES OF THE EYE.

By E. L HOLMES, M. D., of Chicago,

*Lecturer on Diseases of the Eye and Ear in Rush Medical College, and Surgeon of the Chicago Charitable Eye and Ear Infirmary.*

### THE LIDS.

Gentlemen:—Although the lids may be considered simply as organs of protection to the eye, and in themselves scarcely connected with the sense of sight, yet when they are diseased, parts of the eye, vital in importance in the act of seeing, are very liable to suffer. You have already seen many patients, in whom disease of the lids have produced either total or partial loss of vision.

The lids are quite complex in structure. First, there is a very delicate integument, richly supplied with nerves of sensation from the 5th pair. Beneath this is a stratum of cellular tissue, embedded in which are sweat and hair follicles. This layer covers the orbicularis, which is a thin band of muscular fibres, embracing nearly the whole width of the lids. The muscle is thickest near the free edge of the lids. Beneath the muscle is a layer of cellular tissue, quite rich in fat cells. This layer covers the tarsal cartilage, which is especially developed in the upper lid, and which gives firmness and support to it. The lining membrane of the lids has already been described. In the substance of the cartilages are embedded the meibomian glands, which may be seen, on everting the lid, as fine, yellow-colored lines, arranged not unlike certain familiar fern leaves. The minute openings of these glands may be seen along the inner edge of the lids, by the presence of fine drops of secretion pressed out by the effort of turning the lid.

The cilia commence from hair follicles situated quite deep in the lids near the cartilage, and curve upward and forwards in the upper lid, and downward and forwards in the lower lid. They pass through the integument at the outer edge of the lid. At the side of each of the cilia are minute sebaceous follicles, which supply a substance to soften the cilia and the edge of the lids.

All these tissues should be well understood by you. It is important for you to examine, frequently, healthy lids of different individuals and different ages—to observe their thickness, the form of their edges, the relative position of the cilia, the number and size of the folds of skin upon the lids, the position of the puncta lachrymalia and also their movements, whether they close or apen naturally in winking. By doing this frequently you will be able to detect at a glance any abnormal change.

The movements of the lids are influenced by two distinct nerves, a branch of the 3d pair supplying the levator muscle of the upper lid, and a branch of the 7th pair supplying the orbicularis. A paralysis of the former causes an involuntary closure of the muscle, called ptosis; of the latter, an involuntary opening of the lids. Ordinary winking is not under the control of the will, and yet the will can suspend, for a period at least, the motions of the lids. There seems to be special power of the will over the contractions of the orbicularis, in keeping the lids closed.

The lids not only serve as a covering and protection of the eyes, but also assist in the performance of several important functions. By their motions they distribute the lubricating secretions of the conjunctiva and meibomian glands over the surface of the eye, and at the same time remove dust and undue collections of mucus, which may rest upon the cornea. By the motions of the lids also, the tears, in some way not in all respects well understood, are made to pass more readily into the lachrymal sack and nasal duct, than when the lids are at rest.

As it is impossible in a single lecture to describe minutely all the diseases peculiar to the lids, I shall simply draw your attention to some of the most important principles, which may guide you in the more extended study of the subject. It will be convenient to divide these diseases into three classes, those which are usually secondary to some other disease, as for instance, Trichiasis, Entropion, Ectropion, and Adhesions of the lids at the outer angle, all of which are particularly liable to follow chronic conjunctivitis; those tending to the formation of pus, as Simple Abscess, and Hordeolum; 3d, chronic disease of the sebaceous glandulæ.

Trichiasis is a displacement of the cilia, and is almost invariably produced by the atrophy of tissue, resulting from chronic conjunctivitis. It may also be caused by repeated attacks of inflammation of the sebaceous follicles, near the cilia. Wounds of the lids, involving a loss of tissue in the cartilage and conjunctiva, in healing will sometimes cause a contraction of the parts and turn some of the cilia against the globe. In rare instances, even where no disease is apparent, and where the eyes in other respects seem normal, one or more cilia will be found growing from the inner edge of the lids, agasnst the cornea. Mere inspection will enable you in most cases to discover the presence of these misplaced cilia. Sometimes, however, the cilia are so fine and white that great care in an examination is required.

The treatment consists in the destruction of the cilia by introducing a needle covered with caustic, down to the follicle of each misplaced hair; in clipping off a portion of the outer tissue of the lid, near the cilia, the cicatrization of the wound being left to turn the cilia from the globe; and finally, in removing the tissue involving the follicles of the cilia, by making an incision parallel to the edge of the lid, down to the cartilage, and dissecting this strip, care being taken to remove all the follicles without injuring the cartilage. Simple evulsion of the cilia with forceps affords but a temporary relief.

Entropion is that condition in which the integument of the lid is turned against the globe. This unnatural position of the lid produces great irritation and pain, which, sooner or later, causes ulceration and final destruction of the cornea. The disease, in its usual form, is produced by spasmodic contractions of the orbicularis muscle, excited by the irritation accompanying conjunctivitis. The reason why some cases of conjunctivitis are attended with irritation and spasm, not in proportion to the apparent severity of the disease, is not easily explained. The under lid, in consequence of the want of firmness of its cartilage is especially liable to entropion.

The operation for this disease in its uncomplicated form is exceedingly simple, consisting merely in excising one or more pieces from the integument of the lid near its free edge. I usually leave the wound without stitches, to heal by granulation. The contraction produced by cicatrization restores the lid to its natural position. As soon as the wound has sufficiently healed, treatment should be directed to the primary disease. There is scarcely a minor operation in ophthalmic surgery attended so often with satisfactory results as this.

Care should be taken not to remove too much tissue, since the edge of the lid may thus be drawn too far from the globe. Whenever Entropion is complicated with Trichiasis the prognosis is generally less favorable. Although a suitable operation may much improve the condition of the eye, the hardened contracted edge of the lid will often remain a source of irritation.

Ectropion is a disease, usually of the lower lid, in which the conjunctiva is turned from the globe, and exposed to direct contact with the air. It is more frequently a serious disease than Entropion, and may be produced by several conditions of the lids. Total or partial paralysis of the orbicularis muscle may cause it, by permitting the lower lid to fall away by its own weight from the globe. The presence of granulations and hypertrophy of the conjunctiva, by separating the edges of the lid from the globe, gradually tends to produce it. The excoriations of the skin, and consequent loss of tissue and contractions, produced by the presence of abnormal discharges from the eye, is an occasional cause of the disease. The same effect is produced by certain wounds, burns, abscesses, and erysipelas.

The exposure of the conjunctiva and of the cornea to the air, the constant flow of tears upon the cheeks, caused not only by this exposure, but also by the eversion of the punctum, which prevents the tears from passing into the nasal duct, not only render this disease very annoying but, sooner or later, totally or partially destroy vision.

As a satisfactory discussion of the surgical treatment of this disease requires more space than we can at present give, we would refer the readers of the *Journal* to works general surgery as well as those on diseases of the eye.

Chronic Conjunctivitis produces, not unfrequently, by the presence of acrid discharges, excoriations of the edges of the lids at the outer angles. The denuded surfaces thus tend to unite, reducing the length of the palpebral fissure, The irritation of the primary disease, augmented by the abnormal pressure of the contracted lids upon the globe, is increased by spasm of the orbicularis. This condition may be improved in connection with appropriate treatment directed to the primary disease, by making a horizontal incision through the thickness of the lids, at the external angle, thus restoring the palpebral fissure to its normal length. The insertion of two or three vertical stitches, embracing nearly the whole width of the orbicular muscle, will also assist in overcoming spasm and tendency to entropion. The stitches should be drawn quite tight and left to slough out.

Abscess of the lids, whether caused by the inflammation of the minute sebaceous follicles or otherwise, is of comparative little importance, since the tending is almost invariably to recovery without special treatment. And yet there are a few practical points for you to bear in mind. Simple abscess in or near the lids should be treated as abscess in other parts of the body. Hordeolum—sty—is merely suppurative inflammation of the sebaceous follicles. Although it generally requires scarcely any treatment, occasionally there is a long-continued and obstinate tendency to the formation of these abscesses—as in certain cases of repeated occurrence of boils, and treatment seems of little avail. In some of these obstinate cases the use of poultices applied to the lids for a few moments, several times a day, rubbing to the edges of the lids at night a little red precipitate ointment, gr. xvj to the ounce, will prove beneficial. The use of alkalies, in the form of Seidlitz powders, or Rochelle salts, sometimes seems to act favorable. It is well to remember that, now and then there are cases, in which the earliest symptoms are œdema of the conjunctiva, as well as of the lids, presenting the appearance of severe conjunctivitis, and still the disease may be a simple sty, the opening of which at once relieves the œdema. Of course the application of caustic astringents would be worse than useless.

There is another form of abscess, which you will occasionally observe, caused by obstruction and inflammation of the meibomian glands. Usually, however, the disease tends to the formation of cystic tumors, which is of a very slow growth and is filled with an abnormal secretion of the glands.

The tumor may tend to grow at the expense of the tarsal cartilage and conjunctiva, thus forming an elevation upon the inner surface of the lid, or it may press outwards, the tumor appearing upon the outside of the lid. There is but little pain attending the growth of these tumors, although they occasionally become painful and suppurate.

The most reliable treatment consists in opening the tumor and removing as much of the cyst, with its contents, as possible. It may be necessary, sometimes, to destroy portions of the cyst that may remain, by introducing into the wound small portions of nitrate of silver.

Chronic Inflammation of the sebaceous follicles of the lids is quite a common and obstinate disease. It is characterized by the presence of a yellow, hardened secretion upon the edge of the lids, around the base of the cilia. It may involve the whole extent of both lids, or be confined to a few cilia of either lid. A large number of these cases seem to depend upon a scrofulous diathesis. The disease is one of considerable importance, for, although its progress is usually slow, even continuing for years without much change, it is very liable, if neglected, to result in serious injury of the lids.

The irritation produced by the presence of the dried secretions and by the inflammation of the follicles, is extended to the conjunctiva. The disease seems, sometimes, to include an eczematous condition of the edge of the lids. The constant inflammation of the sebaceous follicles, extending to the hair follicles, either tends to destroy the cilia, or to displace them, as in trichiasis. In other cases the inflammation produces great infiltration and hypertrophy of the lid, so that it presents a forbidding appearance, the edge being red and thickened, some of the cilia being totally destroyed, and others being displaced, as in trichiasis. The conjunctiva is also usually inflamed.

The treatment, to be most successful, should be commenced in the early stage of the disease. As the patients often present symptoms of a scrofulous diathesis, particular attention should be paid to the general health. In such cases, pure air, nutritious diet, cod liver oil, iodide of potash, and sometimes, minute doses of bichloride of mercury, are especially indicated. Locally, the eyes should be kept clean during the day, by poultices of starch or slippery elm, applied for a few moments several times a day. During the intervals, the lids should be kept free from collections of secretion by rubbing the edges of the lids with any simple ointment.

Every night an ointment of red precipitate, gr. x—xx to the ounce, should be carefully applied to the edge of the lids. I have found in some obstinate cases that a strong solution of nitrate of silver, ℈j to the ounce, applied by means of a fine camel's hair pencil, to the base of the cilia, will prove beneficial. Tincture of iodine applied in the same way is often very effectual. Great care is required that these last mentioned remedies do not enter the eye.

*Clay as an Application to Ulcers.*—Dr. Schreber, Leipsic, recommends the use of clay as the most innocent, the most simple, and the most economical of palliative applications to surfaces yielding foul and moist discharges. He moreover considers that it has a specific action in accelerating the cure. Clay softened down in water, and freed from all gritty particles, is laid, layer by layer over the affected part to the thickness of about a line. If it becomes dry and falls off, fresh layers are applied to the cleansed surface. The irritating secretion is rapidly absorbed by the clay, and the contact of air prevented. The cure thus goes on rapidly. This clay ointment has a decisive action in cases of fœtid perspiration of the feet or armpits. A single layer applied in the morning will destroy all odor in the day. It remains a long time supple, and the pieces which fall off in fine powder produce no inconvenience.—*Dublin Medical Journal.*

